# Limited impact of an invasive oyster on intertidal assemblage structure and biodiversity: the importance of environmental context and functional equivalency with native species

**DOI:** 10.1007/s00227-018-3338-7

**Published:** 2018-04-20

**Authors:** Nadescha Zwerschke, Philip R. Hollyman, Romy Wild, Robin Strigner, John R. Turner, Jonathan W. King

**Affiliations:** 1 School of Ocean Sciences, Bangor University, Menai Bridge, Anglesey LL59 5AB UK; 2 Centre for Applied Marine Sciences, Bangor University, Menai Bridge, Anglesey LL59 5AB UK

## Abstract

Impacts of invasive species are context dependent and linked to the ecosystem they occur within. To broaden the understanding of the impact of a globally widespread invasive oyster, *Crassostrea* (*Magallana*) *gigas,* intertidal surveys were carried out at 15 different sites in Europe. The impact of *C. gigas* on macro- (taxa surrounding oyster > 1 cm) and epifaunal (taxa on oyster < 1 cm) benthic communities and α and β-diversity was assessed and compared to those associated with native ecosystem engineers, including the flat oyster *Ostrea edulis*. Whilst the effect of *C. gigas* on benthic community structures was dependent on habitat type, epifaunal communities associated with low densities of *O. edulis* and *C. gigas* did not differ and changes in benthic assemblage structure owing to the abundance of *C. gigas* were therefore attributed to the presence of oyster shells. Macrofaunal α-diversity increased with *C. gigas* cover in muddy habitats, while epifaunal α-diversity decreased at greater oyster densities. Macrofaunal β-diversity was greatest at low densities of *C. gigas*; however, it did not differ between samples without and increased densities of oysters. In contrast, epifaunal β-diversity decreased with increasing oyster cover. Different environmental contexts enabled more independent predictions of the effect of *C. gigas* on native communities. These were found to be low and more importantly not differing from *O. edulis*. This indicates that, at low densities, *C. gigas* may be functionally equivalent to the declining native oyster in terms of biodiversity facilitation and aid in re-establishing benthic communities on shores where *O. edulis* has become extinct.

**Electronic supplementary material:**

The online version of this article (10.1007/s00227-018-3338-7) contains supplementary material, which is available to authorized users.

## Introduction

Globalisation and climate change have accelerated the spread of invasive species, which are typically associated with loss of diversity, alterations of ecosystem services and displacement of native species (Vitousek 1990; Vitousek et al. 1996, Butchart et al. 2010; Bellard et al. 2013). Some invasive species are ecosystem engineers and are capable of actively altering the habitat of the recipient communities (Jones et al. 1997; Crooks 2002). The impact of such invasive ecosystem engineers is not always clear, since additional structure and shelter, like those provided by invasive reef builders, affect biotic interactions, biodiversity and other ecosystem processes in different ways (Crooks 2002; Bouma et al. 2009; Katsanevakis et al. 2014; Guy-Haim et al. 2018). The lack of unified methods to identify adverse effects of non-native species, in combination with studies carried out on small localised scales, hampers efforts to generalise predictions of impacts of non-native species over broader spatial scales (Thomsen et al. 2011; Kumschick et al. 2015). Negative impacts of invasive species are often found on small localised scales, but these do not represent spatial variability and context dependency of invasion effects accurately (Fridley et al. 2007; Hulme and Barrett 2013). Surveys and experimental studies on the impact of invasive species are generally driven by different hypotheses within different environmental contexts and thus results are often not comparable (Kumschick et al. 2015). To allow for more ubiquitous predictions to be made, which pinpoint areas of concern and help to more efficiently guide conservation measures, comparable surveys have to be carried out over broader spatial scales incorporating a variety of different environmental contexts (Kumschick et al. 2015).

The Pacific oyster *Crassostrea gigas* (*Magallana gigas*; Salvi and Mariottini 2016; Bayne et al. 2017) has been introduced to Europe, the Americas and Australia as an alternative to declining native oyster fisheries and has since established extensive wild, self-sustaining populations (Eno et al. 1997; Shatkin et al. 1997). Being extremely versatile, *C. gigas* usually occurs within the mid to low intertidal zone over a range of different habitats, such as estuaries, sea loughs and exposed rocky shores in which it creates a novel biogenic reef habitat (Kochmann et al. 2013). It was expected that the spread of *C. gigas* in introduced areas was limited owing to an insufficient number of days at which seawater reaches the range of temperatures (15–25 °C) required for conditioning, larvae survival and settlement of spat (Child and Laing 1998; Syvret et al. 2008). Predicted increases in seawater temperature caused by global warming, however, may increase and accelerate its spread (Rinde et al. 2016; Robins et al. 2017). Areas with dense populations of *C. gigas* report differing consequences of its abundance (Herbert et al. 2016). For example, in Australia, it is predicted that *C. gigas* may displace the native Sydney rock oyster in both mid and low intertidal zones (Krassoi et al. 2008) where it also alters native species assemblages (Wilkie et al. 2012). Within Europe, *C. gigas* is thought to limit the abundance of another ecosystem engineer, the protected honeycomb worm *Sabellaria alveolata* (Dubois et al. 2006; Green and Crowe 2013) and has the potential to displace the native flat oyster *Ostrea edulis* (Laugen et al. 2015; Zwerschke et al., unpublished data). Previously, it was thought that habitats of *O. edulis* and *C. gigas* did not overlap; however, recent surveys show that both species co-occur at similar shore heights (Laugen et al. 2015; Zwerschke et al. 2017). So far, juvenile oyster clusters of both species have been found to support similar species assemblages and diversity on hard substratum (Zwerschke et al. 2016). Yet, it is unclear whether species assemblages and diversity would differ between *C. gigas* and *O. edulis* in different habitat types or in mature oyster beds owing to more pronounced differences in morphology and life history traits of each oyster species (Mann 1979; Green 2017; Nielsen et al. 2017).

Over the last decade, several surveys have been carried out in the NE Atlantic to quantify the impact of *C. gigas* on diversity and benthic assemblage structure (Herbert et al. 2016). One of the more extensive surveys destructively sampled 50 m^2^ oyster reefs on rocky as well as muddy substratum to assess macrofaunal abundance and species richness at two locations on the west coast of France (Lejart and Hily 2011). Oyster reef assemblages were compared to those found on bare substratum of an equal area or within soft sediment and were found to be greater in macrofaunal abundance within *C. gigas* reefs on both rocky and muddy substratum (Lejart and Hily 2011). Additionally, the relative abundance of functional groups was altered by *C. gigas* (Lejart and Hily 2011). In Sweden and the Dutch and German Wadden Sea, experimental studies and surveys were carried out to compare α-diversity and macrofaunal assemblages associated with *C. gigas* reefs to those associated with a native ecosystem engineer, the blue mussel *Mytilus edulis* (Kochmann et al. 2008; Markert et al. 2009; Hollander et al. 2015). Their findings concurred in so far as they found differences in macrofaunal assemblages between both ecosystem engineers, owing to structural differences between oysters and mussels, but differed in the key species driving these shifts (Kochmann et al. 2008; Markert et al. 2009; Hollander et al. 2015).

Impacts of globally established *C. gigas* populations have been widely regarded as potentially harmful to the ecosystem (Herbert et al. 2016). Few studies, however, have aimed to identify a context independent effect of *C. gigas* on assemblage structure and biodiversity by including different geographical regions and ecosystems in their approach. It remains unclear whether the altering degrees of severity of impacts caused by *C. gigas* (Kochmann et al. 2008; Markert et al. 2009; Zwerschke et al. 2016) are owing to different abiotic and biotic conditions or differing methods between studies. In the present study, we aimed to gain a clearer understanding of the impact of *C. gigas* on benthic assemblages as well as α-diversity (species richness and their relative abundance in one community) and β-diversity (diversity between communities; Gray 1997) within different environmental contexts (sensu Padilla 2010) in different geographical regions. Such a comprehensive survey will help to identify context-independent impacts of *C. gigas* on invaded ecosystems that are only visible over a broader scale such as changes in β-diversity (Green and Crowe 2014) or alterations in species distributions across shores. This systematic assessment was carried out at 15 sites across the UK, Ireland and France and it was expected that habitat alterations due to ecosystem engineering capacity of *C. gigas* alters (i) macro and epifaunal assemblage structure within sites by changing the abundance of key species and (ii) biodiversity, regardless of the habitat type. It was further hypothesised that (iii) where *C. gigas* and *O. edulis* co-occurred, epifaunal assemblages supported by both species would differ and (iv) occurrence of *C. gigas* over a broad scale would reduce β-diversity by creating similar biogenic reef habitats across different substratum types.

## Methods

**Fig. 1:**
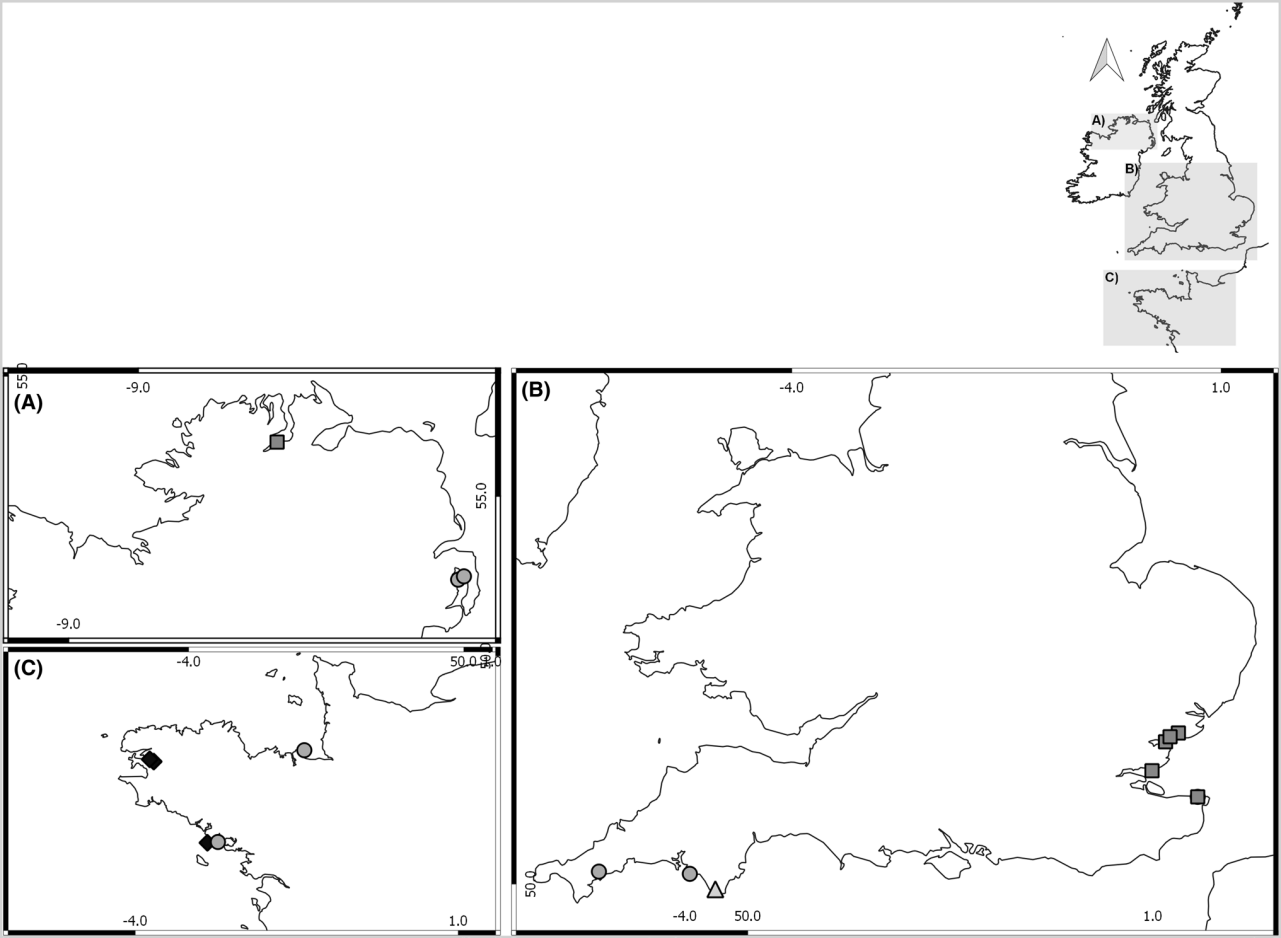
Locations of sites surveyed. Mean oyster abundance at each site was classified into the SACFOR scale (Hiscock 1996; Connor et al. 2004) [superabundant > 100 oysters/m^2^ (black diamond), abundant = 10–99 oysters/m^2^ (dark grey square) common = 1–9 oysters/m^2^ (grey circle) and frequent = 0.1–0.9 oysters/m^2^ (light grey triangle)]

Wild populations of *C. gigas* in Europe occur along a latitudinal gradient between the Shetland islands and the Mediterranean Sea (Herbert et al. 2016; Shelmerdine et al. 2017). In this study, 15 sites across the UK, Ireland and Northern France were visited from May to July 2011 (Fig. 1, Table 1), to represent some of this latitudinal variation (1000 km × 650 km range). Sites were chosen based on Lallias et al. (2015) and local knowledge of oyster populations from a range of stakeholders (see “Acknowledgements”).

**Table 1:** Surveyed sites, their geographical location and physical attributes where the presence of *C. gigas* and *O. edulis* in brackets and italics (mean/m^2^ ± SD) was detected

Location	Site		Height (mean ± SD)	Rugosity (mean ± SD)	Coordinates		*C. gigas* (*O. edulis*) abundance (mean/m^2^ ± SD)	Substratum	Wave exposure
					Latitude	Longitude			
Cornwall, UK	Turnaware Point		1.54 ± 0.31	0.81 ± 0.08	50.2034	− 5.0338	1.29 ± 1.67	Rocky	Medium
Devon, UK	Noss Mayo		1.89 ± 0.25	0.75 ± 0.09	50.3117	− 4.0628	6.14 ± 4.06	Rocky	Medium
Devon, UK	Snapes Point		1.61 ± 0.06	0.76 ± 0.10	50.2396	− 3.7600	0.86 ± 0.80	Rocky	Medium
Essex, UK	Bradwell		0.76 ± 0.10	0.82 ± 0.04	51.7347	0.8860	74.57 ± 52.06	Muddy	Low
Essex, UK	Brightlingsea		0.49 ± 0.25	0.76 ± 0.05	51.8062	1.0164	52.29 ± 20.70	Muddy	Low
Essex, UK	Southend on Sea	LS	0.26 ± 0.03	0.94 ± 0.03	51.5235	0.7760	32.86 ± 31.05	Muddy	Low
Essex, UK	Southend on Sea	HS	1.70 ± 0.03	0.99 ± 0.01	51.5235	0.7760	23.43 ± 15.65	Gravel	Low
Essex, UK	West Mersea		2.16 ± 0.51	0.82 ± 0.05	51.7734	0.9295	76.57 ± 64.35	Muddy	Low
Brittany, France	Cancale		2.21 ± 1.28	0.74 ± 0.08	48.7022	− 1.8453	5.86 ± 4.80	Rocky	High
Brittany, France	Le Faou		0.42 ± 0.73	0.92 ± 0.03	48.2966	− 4.2197		Muddy	Low
Brittany, France	Moulin-Mer		1.46 ± 0.86	0.80 ± 0.08	48.3120	− 4.2910	248.86 ± 102.53	Rocky	Low
Brittany, France	Penthievre		3.21 ± 0.23	0.83 ± 0.03	47.5419	− 3.1333	117.43 ± 88.61	Rocky	High
Brittany, France	St. Philibert		0.21 ± 0.23	0.99 ± 0.00	47.5703	− 2.9705	4.57 (*3.86*) ± 3.49 (*2.25*)	Gravel	Low
Kent, UK	Birchington	LS	0.74 ± 0.05	0.90 ± 0.01	51.3819	1.3157	32.29 ± 22.79	Mussel bed	Medium
Kent, UK	Birchington	HS	2.18 ± 0.09	0.85 ± 0.04	51.3819	1.3157	2.43 ± 1.94	Rocky	Medium
Donegal, Ireland	Lough Swilly	LS	0.25 ± 0.07	0.93 ± 0.01	55.0209	− 7.5774	7.00 (*1.57*) ± 2.76 (*1.98*)	Gravel	Low
Donegal, Ireland	Lough Swilly	HS	0.74 ± 0.09	0.93 ± 0.01	55.0209	− 7.5774	38.00 ± 10.90	Mussel bed	Low
Down, UK	Mount Stewart		0.64 ± 0.25	0.93 ± 0.03	54.5418	− 5.6044	1.57 (*0.53*) ± 1.72 (*0.49*)	Gravel	Low
Down, UK	Paddies Point		0.70 ± 0.31	0.94 ± 0.05	54.5178	− 5.6504	1.43 (*0.29*) ± 2.00 (*0.49*)	Gravel	Low

Mean abundance of *C. gigas (O. edulis)* was calculated using data collected from all quadrats (control and those containing *C. gigas*) within each shore. If two distinct oyster populations were found at high (HS) and low (LS) positions on the shore, both were sampled as independent sampling events. The numbers in italic and brackets denote densities of the second oyster species *O. edulis*

### Shore survey

Ecological surveys over a variety of habitats are necessarily associated with a high degree of biological and abiotic variability. To capture abiotic factors most likely to drive differences in oyster abundances and assemblage structure, each site was assessed in a similar manner upon arrival on the shore. The type of substrate at each site was classified into four categories (mud, rock, gravel/boulders and mussel bed). Substratum type and abundance of dominant species were used to categorise wave exposure for individual sites (low, medium, high) according to the exposure scale by Ballantine (1961). The shore height above Chart Datum at which the oysters were present was estimated using a surveyor’s level (Leica NA 820, *n* = 5); the maximum and minimum heights on the shore at which oysters were found were also measured. The topography of each site was quantified using rugosity measurements whereby a 5 m chain (*L*_g_) was laid over the substrate, taking into account all fluctuations in surface level and measuring the resulting straight line distance that was covered by the chain (*L*_r_) (Nic et al. 1997). Rugosity was then calculated according to the following equation:

$$f_\text{r} = \frac{{L_\text{r}}}{{L_\text{g}}}.$$

A total of six rugosity measurements were taken at each site, three parallel to and three perpendicular to the shoreline.

### Oyster density and macrofauna

To efficiently assess potential interaction between *C. gigas* densities and macrofaunal assemblages and biodiversity, 30 50 × 50 cm photo-quadrats were recorded on each site, placed randomly within the identified *C. gigas* zone and photographed (Nikon D90). Percentage abundance and presence of all species visible on the photograph of the quadrat (generally all species > 1 cm) were quantified. Abundance of *O. edulis* was limited on shores and was only rarely found in random samples. Thus, to ensure a consistent baseline to which macrofaunal assemblages associated with *C. gigas* could be compared to across sites, we recorded an additional ten haphazardly placed photo-quadrats in areas between *C. gigas* clumps that, purposefully, did not contain any non-native oysters on every site (*N*_control quadrats/site_ = 13.5 ± 9.2 mean ± SD). On sites with distinctly separated *C. gigas* populations at high and low intertidal areas, both zones were sampled as two independent sampling events to accurately represent the associated species assemblages.

### Epifauna

To estimate interaction between *C. gigas* and species living on and within the oyster shell (individuals < 1 cm), 50 oysters were randomly collected from each shore and stored in cool boxes, thereby also preventing the possible loss of any mobile epifauna. Within 24 h of collection of oysters, epifaunal assemblages were sampled by firstly removing mobile organisms with pressurised freshwater over a 500 μm sieve and secondly removing remaining species manually with tweezers. Individual oysters were then photographed (front and back) for subsequent identification of sessile epifauna, which could not be sampled without destroying the organism. All epifaunal species collected from all 50 oysters were pooled in one sample which was analysed for species presence and abundance. Whenever possible, species assemblages and abundances associated with other ecosystem engineers from the same site such as, *O. edulis* and the blue mussel *M. edulis* were used as control. On sites where native European oyster, *O. edulis* populations were present (Paddies Point, Mount Stewart, Lough Swilly and St. Philibert, Table 1), the sampling procedure for epifauna was repeated as outlined above with 50 *O. edulis*. Owing to a severe decline of *O. edulis* populations in recent years, we could only locate the native oyster at these four locations. Only two sites were identified where *C. gigas* was present in *M. edulis* beds (Birchington and Lough Swilly). Here, a 30 × 30 cm quadrat was placed haphazardly over the mussel community without oysters. The mussels contained within the quadrat were removed and treated the same way as the oysters to estimate abundance and identify all associated epifaunal species. The amount of mussels sampled corresponded to the approximate amount of three-dimensional structures provided by the 50 sampled oysters. All epifaunal samples were preserved in 70% IMS and later identified to the lowest possible taxonomic level.

### Image analysis

Photo analysis was undertaken in Image J (Ferreira and Rasband 2011) where percentage cover and presence of each species in the image was recorded for macrofaunal (quadrats) and epifaunal (oysters) analysis, respectively. The abundance of *C. gigas,* estimated from photo-quadrats, was standardised to mean oyster abundance/m^2^ and categorised using the SACFOR scale (superabundant > 100 oysters/m^2^, abundant = 10–99 oysters/m^2^, common = 1–9 oysters/m^2^, frequent = 0.1–0.9 oysters/m^2^, occasional = 0.01–0.09 oysters/m^2^ and rare < 0.009 oysters/m^2^; Hiscock 1996; Connor et al. 2004). This was done for individual photo-quadrats (analysis of macrofauna: number of oysters in a quadrat) and for each sampled site (analysis of epifauna: number of oysters in all 30 quadrats/m^2^ covered by sampled quadrats). Because oysters were either not present or were present in numbers ≥ 1 in photo-quadrats, the abundance of oyster for macrofaunal analysis could not be classified as lower than common. Unfortunately, adequate image analysis was not possible at one site, Le Faou in Brittany, France, owing to the high abundance of *Ulva lactuca* and *Porphyra* sp., which covered benthic assemblages completely and would have caused an inaccurate assessment of benthic assemblage structure.

### Statistical analysis

For statistical analysis, *C. gigas* was not included as a response variable to avoid confounding independent and dependent variables (Huston 1997). Permutational analysis of variance (PERMANOVA; McArdle and Anderson 2001) was used to assess the impact of oyster abundance [macrofauna: absent (*C. gigas* was not present in quadrat), common, abundant, superabundant; epifauna: control (species assemblages collected from *O. edulis* and *M. edulis*), frequent, common, abundant, superabundant], habitat type (rock, gravel/boulders, mussel bed and mud) and wave exposure (low, medium, high) on both macro- and epifaunal assemblages. Factors were nested in sampling sites to account for dependencies of replicates from the same shore. Multivariate analysis were based on Bray–Curtis similarity matrices calculated from untransformed and fourth-root-transformed data to distinguish between the effects of rare and dominant species (Clarke and Warwick 2001) and was carried out under a reduced model with 9999 permutations of the residuals. The robustness of PERMANOVA has been shown to be affected by heterogeneity of multivariate dispersions in combination with an unbalanced sampling design, e.g. a varying amount of replicates for each factor combination (Online Resource 1), in which case a greater tendency to type I errors was observed (Anderson and Walsh 2013). Multivariate heteroscedasticity within explanatory variables was tested for by using the function betadisper (R-package vegan; Oksanen et al. 2017). Unfortunately, multivariate dispersion was not homogenous and could not be altered by applying transformations to either macrofaunal or epifaunal datasets. Owing to a lack of an alternative multivariate test, we proceeded with the analysis in PERMANOVA, but altered our α-level to a more conservative *P* = 0.01. PERMANOVA was also used to test for differences in epifaunal species assemblages associated with *O. edulis* and *C. gigas* at the four sites where both oysters co-occurred in the intertidal zone (Paddies Point, Mount Stewart, Lough Swilly and St. Philibert). Here, the factors oyster species (*O. edulis* and *C. gigas*) and oyster abundance were again nested in sampling site. Pairwise post hoc tests, using a Bonferroni correction, were carried out to differentiate between treatments within significant factors.

Similarity of percentage (SIMPER) analysis was used to identify the most important taxa driving differences between groups of significant factors such as oyster abundance and habitat type. A 90% cutoff was used for the cumulative dissimilarity between groups, because thereafter remaining taxa individually contributed to < 2% of overall differences in assemblage structure. Differences in the abundances of these taxa were analysed with a generalised linear mixed model (GLMM) using penalised quasi-likelihood (PQL) to estimate interference and fitted with a normal or lognormal distribution as dictated by data (Breslow 2003). Factors shown to be significant in the PERMANOVA analysis, such as oyster abundance and habitat type for macrofauna, were added as fixed factors, while sampling site was added as random factor to account for nested data. Least-square means, where *P*-values were adjusted for the Tukey method, were applied as post hoc test to differentiate between significant terms (Lenth 2016).

The Shannon–Weaver index was used to calculate α-diversity for macro- and epifauna. The most appropriate factors describing distribution of diversity were chosen by including all available factors (oyster abundance, habitat type, exposure, shore rugosity, height on shore, latitude) in a linear model and randomly dropping interactions and factors from the model and comparing it to the original model using *P*-values of ANOVA (analysis of variance; Zuur et al. 2009). The final model was tested using GLMM with PQL to estimate interference and was fitted with a normal distribution. For macrofaunal diversity, the fixed factors oyster abundance, habitat type and exposure and the random factor sampling site were included in the analysis. For epifaunal diversity, only the fixed factors oyster abundance and habitat type were included in the analysis. Here, latitude was included as random factor to account for spatial variability and nestedness of samples from similar areas, since replication of epifaunal diversity per site was low [1 factor combinations/site (2, when control values were included)]. To test for the effect of *C. gigas* on epifaunal diversity, α-diversity associated with *O. edulis* and *M. edulis* beds was included in the analysis as control variable. To clearly differentiate whether alternating effects of oyster species on biodiversity exist, a subsequent analysis, limited to the four sites were *O. edulis* and *C. gigas* co-existed, tested for the effect of oyster identity and oyster abundance on diversity, including site as random factor. Homogeneity of variance and normal distribution of residuals from each model were assessed visually to guarantee that data fit the model (Zuur et al. 2009). Least-square means, where *P*-values were adjusted for the Tukey method, were applied as post hoc test to differentiate between significant terms.

To estimate whether β-diversity changes with increasing oyster abundance, local contribution to β-diversity (LCBD) or uniqueness of samples was calculated using the adespatial package on macrofaunal and epifaunal species abundance data (Dray et al. 2017). Differences in LCBDs caused by oyster abundance were then analysed with a GLMM using PQL to estimate interference, fitted with a lognormal distribution. For macrofaunal analysis the fixed factor latitude and the random factor site were included to account for spatial variability and nested data. Owing to low replication, epifaunal analysis only included the random factor latitude to account for nested data along the spatial gradient. All data analyses were carried out in R version 3.4.1 (R Core Team 2017).

## Results

### Effects of *C. gigas* on macro- and epifaunal assemblages

**Fig. 2:**
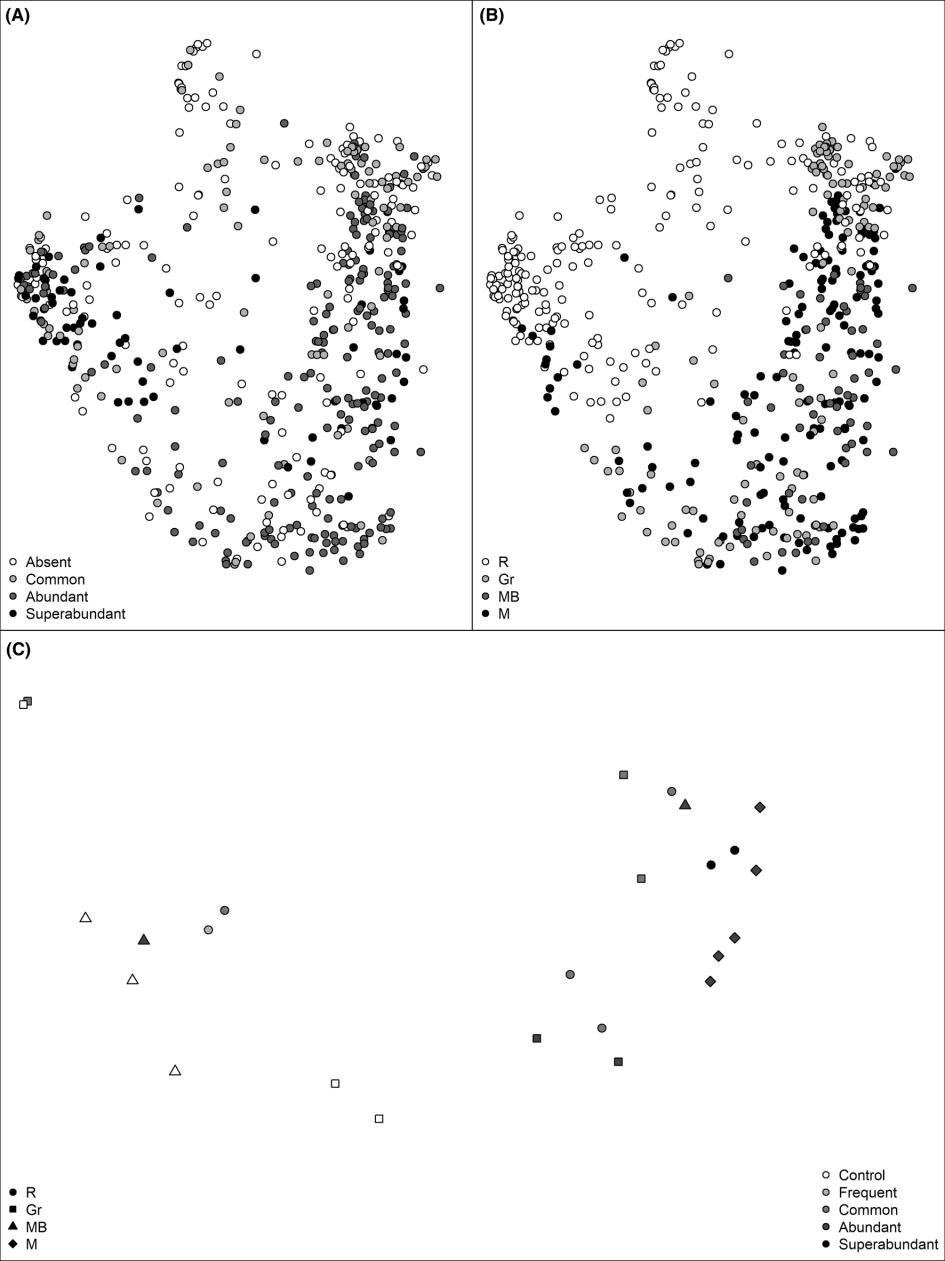
Multi-dimensional scaling (MDS) plot of macrofaunal assemblages at all surveyed sites by (**a**) SACFOR scale and (**b**) habitat type: rocky (R), gravel (Gr), mussel bed (MB) and muddy (M). Oyster abundance (SACFOR) and habitat type for (**c**) epifaunal assemblages are represented by colour and different symbols, respectively. Epifaunal diversity for control treatments was calculated from pooled *M. edulis* and *O. edulis* samples. Macrofaunal diversity for the category “Absent” was calculated from samples that did not contain any *C. gigas*

A total of 74 taxa were identified in macrofaunal assemblages and 295 in epifaunal assemblages. Macrofaunal assemblages showed a significant interaction between oyster abundance and habitat type (Table 2a, Fig. 2a, b). Post hoc tests were inconclusive but provided an indication that at muddy, rocky and sites with gravel, densities of oysters > 10/m^2^ featured a different macrofaunal assemblage structure than those with lower or no *C. gigas* abundance, while in mussel beds assemblage structure only differed when *C. gigas* was common (1–9/m^2^) or abundant (10–99/m^2^). Analysis on fourth-root-transformed data showed a similar interaction between oyster abundance and habitat type (PERMANOVA, *F* (8538) = 4.95, *P* < 0.001); hence, shifts in macrofaunal assemblages are due to shifts of whole communities rather than few dominant species. Taxa driving these differences, as shown by SIMPER analysis, were a greater abundance of barnacles, red algae *Chondrus crispus* and the kelp *Fucus vesiculosus* in plots with abundant to superabundant densities of oysters. In contrast, the blue mussels *M. edulis* and the green algae *U. lactuca* occurred increasingly within plots of no or common presence of oysters (Online Resource 2, 3). The periwinkle *Littorina* sp., however, was present in high densities when oysters were superabundant, common or absent, but only in lower densities when oysters were abundant. Analysis on single species found that barnacles and *F. vesiculosus* showed interactions between habitat types and oyster abundances (Table 3). For barnacles this was also caused by differences in barnacle abundance between habitats, but mostly by differences within mussel beds, where the greatest barnacle abundance was found at oyster densities > 99/m^2^ (Table 3, Online Resource 3). Post hoc tests on *F. vesiculosus* were inconclusive, yet showed a tendency for kelp abundance to increase with oyster abundance in all habitats except rocky shores where it decreased (Table 3, Online Resource 3). Increased densities of oysters also seemed to increase the abundance of *Littorina* sp. in all habitats except rocky shores, with the greatest abundance of periwinkles on sites with oyster density > 99/m^2^ (Table 3, Online Resource 3). Specific analysis on *M. edulis*, *U. lactuca* and *C. crispus* revealed no impact of oyster abundance or habitat type on the distribution of these species (Table 3; Online Resource 3).

**Table 2:** Permutational multivariate analysis of variance (PERMANOVA) testing for the effects of oyster abundance (SACFOR), habitat type and exposure on (a) macrofaunal and (b) epifaunal assemblages

	*Df*	*F*	*R* ^2^	*P*
(a) Macrofauna				
Oyster abundance = OA	3	12.51	0.05	0.665
Habitat = H	3	31.86	0.13	0.019
Exposure = E	2	21.32	0.06	0.999
OA × H	8	4.24	0.05	**< 0.001**
OA × E	4	2.26	0.01	0.990
H × E	1	4.68	0.01	0.388
Residuals	517			
Total	538			
(b) Epifauna				
Oyster abundance	4	2.67	0.30	0.012
Habitat	3	1.99	0.17	0.189
Exposure	2	1.64	0.09	0.107
OA × H	1	1.35	0.04	0.439
OA × E	2	1.23	0.07	0.299
Residuals	12			
Total	24			

Significant results (*P* < 0.01) are presented in bold

**Table 3:** GLMM testing for the effect of oyster abundance and habitat type on the distribution of key species causing a shift in macrofaunal assemblages

	*Df*	*F*	*P*
**Cirripedia**			
Intercept	1	78.97	< 0.0001
Oyster abundance = OA	3	50.77	**< 0.0001**
Habitat = H	3	0.87	0.482
OA × H	9	2.03	**0.035**
***Littorina*** **sp**.			
Intercept	1	22.08	< .0001
Oyster abundance	3	4.85	**0.003**
Habitat	3	0.31	0.818
OA × H	9	0.93	0.499
***Mytilus edulis***			
Intercept	1	47.29	< 0.0001
Oyster abundance	3	1.68	0.171
Habitat	3	0.04	0.990
OA × H	9	0.79	0.625
***Fucus vesiculosus***			
Intercept	1	19.57	< .0001
Oyster abundance	3	0.38	0.771
Habitat	3	2.28	0.124
OA × H	9	3.28	**0.001**
***Chondrus crispus***			
Intercept	1	13.02	0.001
Oyster abundance	3	0.43	0.734
Habitat	3	0.61	0.622
OA × H	9	1.44	0.166
***Ulva lactuca***			
Intercept	1	9.48	0.002
Oyster abundance	3	1.40	0.243
Habitat	3	2.43	0.108
OA × H	9	0.97	0.468

Sampling site was included in the analysis as random factor. Significant effects are presented in bold (*P* < 0.05)

Epifaunal assemblages did not differ between *C. gigas* and *O. edulis* (GLMM, *F* (1, 7) = 0.82, *P *= 0.25) and their abundance (GLMM, *F* (1, 7) = 1.98, *P *= 0.17). Moreover, epifaunal assemblages did not differ with an increased abundance of *C. gigas* or between habitat type or wave exposure (Table 2b, Fig. 2c). Although abundance of *C. gigas* was on the verge of significance, analysis on fourth-root-transformed data fortified the findings of the original analysis, with no shift in assemblages associated with oyster abundance (GLMM, *F* (4, 25) = 1.88, *P *= 0.046), habitat type (GLMM, *F* (3, 25), *P *= 0.27), or wave exposure (GLMM, *F* (2, 25) = 0.99, *P *= 0.22) at our α-level of 0.01.

### Impacts on α-diversity caused by *C. gigas*

**Fig. 3:**
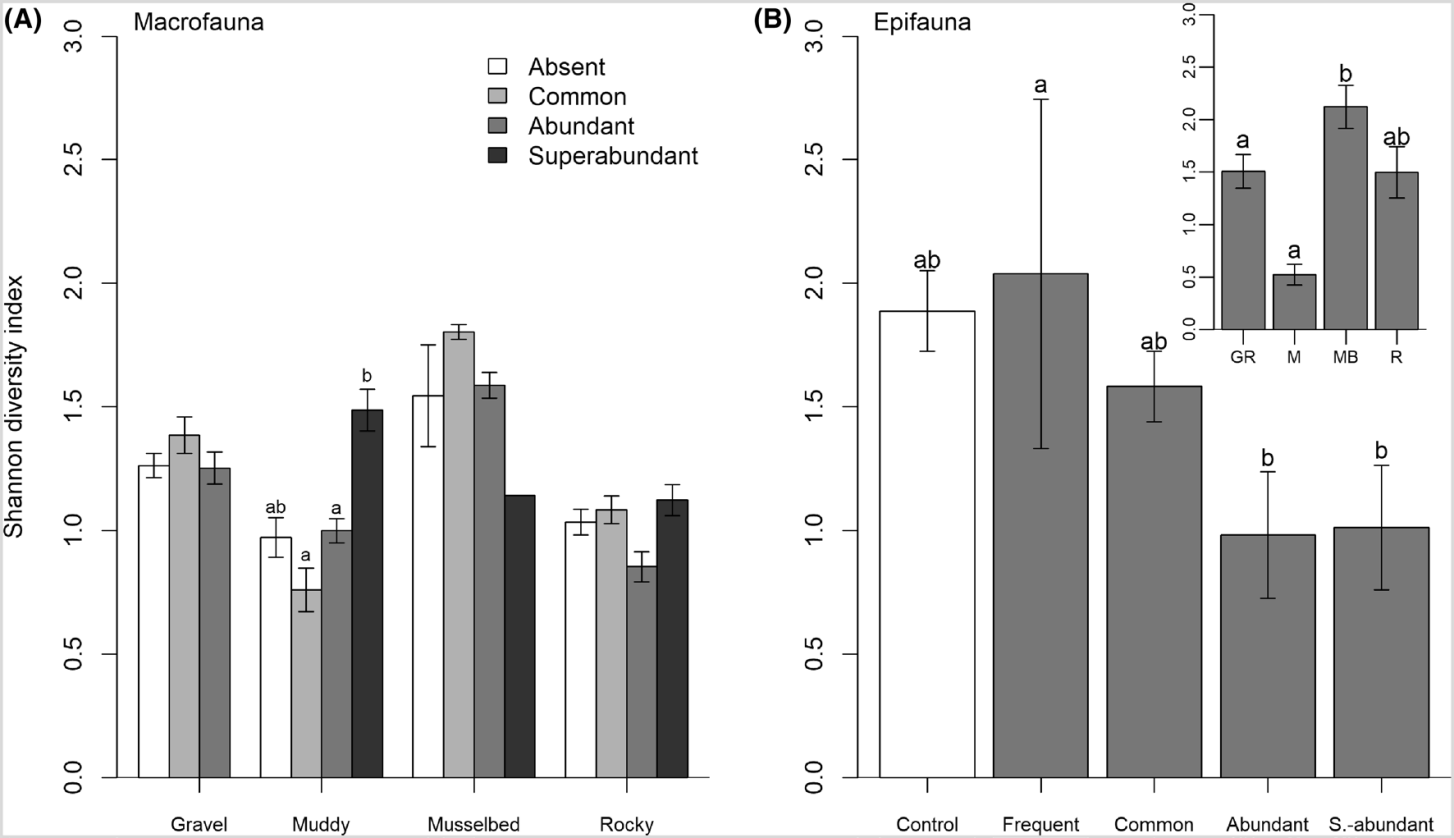
Shannon diversity index (mean ± SD) for (**a**) macrofauna and (**b**) epifauna by oyster abundance and habitat type according to the SACFOR scale. Samples with no *C. gigas* present (absent or control) are represented in white, and samples with *C. gigas* present in grey. Epifaunal diversity for control treatments was calculated from pooled *M. edulis* and *O. edulis* samples. Macrofaunal diversity for the category “Absent” was calculated from images that did not contain any *C. gigas* but might have contained native ecosystem engineers. Inset depicts epifaunal diversity associated with the different habitat types: gravel (GR), mud (M), mussel beds (MB) and rocky shores (R). Error bars represent standard deviations. Different lowercase letters denote significant differences between habitat types and oyster densities

Macrofaunal diversity showed a significant interaction between oyster abundance and habitat type (GLMM, *F* (9, 538) = 2.28, *P* = 0.016; Fig. 3a), yet was not dependent on wave exposure (GLMM, *F* (2, 538) = 2.89, *P* = 0.094). Post hoc tests showed that within muddy habitats, diversity associated with oyster densities > 99/m^2^ was higher than for lower oyster densities (1–99/m^2^). Epifaunal diversity was equally affected by increased densities of *C. gigas* (GLMM, *F* (4, 25) = 9.06, *P* = 0.001) and habitat type (GLMM, *F* (3, 25) = 8.88, *P* = 0.002; Fig. 3b). Here, sites with an oyster density between 0.1 and 0.9 oysters/m^2^ showed greater diversity than those with oyster densities > 10/m^2^, but did not differ from diversity associated with mussel beds and *O. edulis* (Fig. 3b). Significant differences in epifaunal diversity found between habitat types can be mainly attributed to the high epifaunal diversity in mussel beds compared to those with soft or gravely substratum. Most importantly, subsequent analysis on epifaunal diversity associated with *C. gigas* and *O. edulis* showed no difference between the two species (GLMM, *F* (1, 7) = 1.8, *P* = 0.27) at different oyster abundances (GLMM, *F* (1, 7) = 5.25, *P* = 0.15).

### Effect of *C. gigas* on β-diversity

For macrofaunal assemblages, uniqueness of site was greater in plots where oysters were common (1–9/m^2^) than in plots were *C. gigas* was absent, however neither differed from sites with greater oyster densities (GLMM, *F* (3, 538) = 3.19; *P* = 0.023; Online Resource 4). Uniqueness of sites for macrofaunal assemblages also decreased with increasing latitude (GLMM, *F* (1, 538) = 5.45; *P* = 0.033). In contrast, epifaunal assemblages became more similar on sites with oyster densities > 99/m^2^ (GLMM, *F* (4, 20) = 3.37; *P* = 0.037, Online Resource 4).

## Discussion

The globally abundant oyster *C. gigas* is classified as invasive in many regions in the NE Atlantic, such as in the Wadden Sea and within the UK (Nehring 2006; Dutertre et al. 2010; Global Invasive Species Database 2015). Until now, its impacts have not been classified by a systematic sampling design, incorporating context dependency and spatial scale. The current survey shows clearly that impact of *C. gigas* on macrofaunal assemblages is highly context dependent and changes with different oyster densities at different habitat types. On rocky shores for example, assemblages in plots with oyster densities > 10 m^2^ differed from plots containing less or no oysters in mussel beds; however, differences in macrofaunal assemblage structure were only perceived between plots where oysters were common or abundant. Impacts of *C. gigas* on macrofaunal diversity were restricted to muddy habitats, where diversity increased with increasing oyster density. Epifaunal assemblages were not affected by the presence of *C. gigas* compared to assemblages associated with *O. edulis* and *M. edulis*; epifaunal diversity, however, was highest at low densities of *C. gigas* and decreased with increasing densities. Similar epifaunal assemblages and biodiversity associated with low densities of *C. gigas* and *O. edulis* suggest functional equivalence of the two species in terms of habitat provision and biodiversity facilitation. Interestingly, while intermediate oyster density increased macrofaunal β-diversity, epifaunal species assemblages in different environmental regions showed an indication of becoming less unique with increasing *C. gigas* abundance.

The potential of *C. gigas* to alter species assemblages has been already widely known (Kochmann et al. 2008; Markert et al. 2009; Padilla 2010; Green and Crowe 2014; Norling et al. 2015). Comparisons with other native habitat engineers, such as the blue mussel *M. edulis*, have shown that these changes are dependent on the structural properties of the ecosystem engineer (Buschbaum et al. 2006; Gain et al. 2017). Similarities in epifaunal assemblages associated with both *C. gigas* and *O. edulis* support these findings and emphasise the importance of the unique biogenic habitat provided by oysters. These findings are also supported by a recent experiment comparing benthic assemblages associated with *C. gigas* and *O. edulis* at intertidal and subtidal hard substratum habitats (Zwerschke et al. 2016). In the past, most European shores would have included dense populations of native oysters *O. edulis* (Riesen and Reise 1982; Reise et al. 1989) and their associated benthic assemblages (Smyth and Roberts 2010). The recent decline of *O. edulis* populations resulted in altered benthic assemblage structure on these shores. The occurrence of wild *C. gigas* populations, albeit potentially harmful to *O. edulis* itself (Zwerschke et al., unpublished data), may help to re-establish coastal benthic communities to a former state. Here, we have increased the scope of previous understanding with regard to functional similarities between *C. gigas* and *O. edulis* and show that both oyster species support similar communities under varying environmental context when naturally co-occurring.

The effect of *C. gigas* differs between macrofaunal and epifaunal diversity and appears to be highly context dependent on habitat type. We suggest that the structural properties of the oysters were most important in soft sediment habitats for macrofauna and were underpinning the decrease in epifaunal diversity at increasing oyster densities. This is in concurrence with the theory that the greatest diversity is generally found at an intermediate level of structural complexity and that increased structural complexity reduces diversity, owing to a lower availability of settlement space (Snover and Commito 1998; Allouche et al. 2012). A similar pattern has often been observed for other ecosystem engineers as well. For example, an increase in structural complexity by densely packed turf algae reduces associated gastropod diversity (Kelaher 2003). The fact that diversity was similar between *C. gigas* and *O. edulis* raises the question whether diversity associated with formerly dense *O. edulis* beds would have followed a similar pattern. Unfortunately, low abundance of *O. edulis* did not allow for direct comparison of epifaunal diversity associated with both species for every abundance category. It is notable, however, that another study in Australia found similar epifaunal species richness associated with *C. gigas* and the native *Saccostrea glomerata* at different densities (Wilkie et al. 2012). Therefore, we suggest that the decline in epifaunal diversity associated with an increase in oyster density is the result of the decreased abundance of settlement space and an increased habitat fragmentation associated with such dense assemblages, which also occurs in other native ecosystem engineers (Tokeshi and Arakaki 2012).

Interactions between *C. gigas* and other key species seem to alter with habitat type. The abundance of barnacles for example increased with increasing oyster cover on rocky shores and mussel beds, but was greatest at low oyster densities at muddy sites and subsequently declined with increasing oyster cover. Furthermore, the abundance of *F. vesiculosus* and *Littorina* sp. generally increased with increasing oyster densities in all habitats, with the exception of rocky shores where their abundance decreased. Other species interaction may play a major role in determining such species abundance patterns. For example, is it likely that mechanisms interfering with successful recruitments, such as propagule pressure and abundance of grazers disturbing recently settled barnacle larvae or algae seedlings, may vary with time and location, while biological interactions may change with the maturity of oyster reefs (Jenkins et al. 2000; Holmes et al. 2005; Rezek et al. 2017). High densities of *C. gigas* may also limit the initial abundance of early colonizers such as barnacles possibly by ingestion of larval stages (Troost et al. 2008), but may prove to be a refuge from predation through the provision of complex three-dimensional structure for matured communities, thereby causing an accumulation of individuals over time (Grabowski 2004).

The spread of invasive species has often been linked to the loss of β-diversity along spatial scales (McKinney and Lockwood 1999; Piazzi and Balata 2007). Invasive ecosystem engineers, such as *C. gigas*, can cause a reduction in habitat heterogeneity by creating a similar habitat (e.g. oyster reefs) across different substratum types. This may subsequently lead to a reduced availability of habitat types, thereby decreasing β-diversity of associated communities (Piazzi and Balata 2007). To our knowledge, this was the first time the impact of *C. gigas* on β-diversity was observed over a large scale. When oysters were common, their abundance in different habitats may have contributed to a greater habitat heterogeneity by providing more and varying types of three-dimensional structure in the ecosystem, thus increasing β-diversity (Bouma et al. 2009). In contrast, greater oyster densities may reduce habitat heterogeneity, by providing a consistent three-dimensional space across different substratum types, which may have contributed to the observed decrease in epifaunal β-diversity (Bouma et al. 2009). A separate study found that high densities of *C. gigas* (240 oysters/m^2^) can reduce β-diversity on a local scale (Green and Crowe 2014) and it is likely that the uniform habitat structure, created by such densities of *C. gigas*, attracts similar species assemblages and contributes to this loss of β-diversity. It must be borne in mind, however, that densities of *C. gigas* usually vary greatly across shores. In general, it is uncommon for *C. gigas* to populate entire shores and it is most likely that the oyster will be found in varying densities in a band between the mid and low intertidal zone (Kochmann et al. 2013). It is also unclear whether a similar β-diversity reducing effect can be observed for other ecosystem engineers populating different habitat types across Europe, such as the blue mussel *M. edulis*, and whether a similar reduction of β-diversity was associated with *O. edulis* when it used to be present in greater densities.

Including context dependency in surveys to classify the impacts of invasive species is extremely important, since the impacts may vary in different habitats, with different abiotic factors and different biotic interactions (Thomsen et al. 2011; Green and Crowe 2014; Kumschick et al. 2015). The present study is one of the few, spanning a variety of different habitat types and environmental conditions, which allows us to deduct more general predictions of the impact of *C. gigas* on native benthic assemblages and diversity. The most visible impact of *C. gigas* was limited to soft substratum habitats and areas with high densities of *C. gigas*. Here, we suggest that alterations of benthic community structure and diversity caused by the presence of *C. gigas* are owing to the provision of the unique habitat that is being created by oyster shells and is not specific to different oyster species. We propose that the presence of *C. gigas* will provide a habitat suitable for benthic communities formerly associated with declining *O. edulis* populations. In light of the global decline of ecologically valuable oyster reefs (Beck et al. 2011), and current restoration efforts (Laing et al. 2006; Lallias et al. 2010; Gercken and Schmidt 2014), the here observed functional similarity between low densities of *O. edulis* and *C. gigas* with regard to habitat provisioning may initiate a reconsideration of conservation goals in Europe. If the main objective is to create a three-dimensional structure that supports diversity and assemblages which are unique to oyster reefs, it may be worth to consider already abundant *C. gigas* populations as a valuable alternative to a more problematic re-introduction of *O. edulis* on European shorelines.

## Electronic supplementary material

Below is the link to the electronic supplementary material.

Supplementary material 1 (PDF 203 kb)

Supplementary material 2 (PDF 249 kb)

Supplementary material 3 (PDF 105 kb)

Supplementary material 4 (PDF 193 kb)
