# Supplementary material for: Limited impact of an invasive oyster on intertidal assemblage structure and biodiversity: the importance of environmental context and functional equivalency with native species
Source: Mar Biol. 2018 Apr 20;165(5):89. doi: 10.1007/s00227-018-3338-7 (PMC5910461; doi:10.1007/s00227-018-3338-7)
Supplement: Supplementary file 1 — Supplementary material 1 (PDF 203 kb) [file 227_2018_3338_MOESM1_ESM.pdf]

Marine Biology

Limited impact of an invasive oyster on intertidal assemblage structure and biodiversity: The importance of environmental context and functional equivalency with native species

Nadescha Zwerschke[1]*, Philip R. Hollyman[1], Romy Wild[1], Robin Strigner[1], John R. Turner[1], Jonathan W. King[2]

[1]School of Ocean Sciences, Bangor University, Menai Bridge, Anglesey, LL59 5AB, UK

[2] Centre for Applied Marine Sciences, Bangor University, Menai Bridge, Anglesey, LL59 5AB, UK

Corresponding author: nzwerschke01@qub.ac.uk

Supplementary Material 1: Number of replicates available for multivariate analysis of a) macrofaunal and b) epifaunal assemblages for calculated factor interactions.

A) Macrofauna

		Absent	Common	Abundant	S.-Abundant	High	Medium	Low
Exposure	High	11	15	19	15			
	Medium	75	45	29	1			
	Low	73	52	154	50			
Habitat	Rocky	84	55	28	43	60	120	30
	Gravel	51	33	35	0	0	0	119
	Mussel	4	5	50	1	0	30	30
	Muddy	20	19	89	22	0	0	150

B) Epifauna

		Absent	Frequent	Common	Abundant	S.-Abundant	High	Medium	Low
Exposure	High	0	0	1	0	1			
	Medium	1	1	3	1	0			
	Low	5	0	3	8	1			
Habitat	Rocky	0	1	3	0	2	2	4	1
	Gravel	3	0	4	2	0	0	0	8
	Mussel	3	0	0	2	0	0	2	3
	Muddy	0	0	0	5	0	0	0	5
