# Supplementary material for: Limited impact of an invasive oyster on intertidal assemblage structure and biodiversity: the importance of environmental context and functional equivalency with native species
Source: Mar Biol. 2018 Apr 20;165(5):89. doi: 10.1007/s00227-018-3338-7 (PMC5910461; doi:10.1007/s00227-018-3338-7)
Supplement: Supplementary file 2 — Supplementary material 2 (PDF 249 kb) [file 227_2018_3338_MOESM2_ESM.pdf]

Marine Biology

Limited impact of an invasive oyster on intertidal assemblage structure and biodiversity: The importance of environmental context and functional equivalency with native species

Nadescha Zwerschke[1]*, Philip R. Hollyman[1], Romy Wild[1], Robin Strigner[1], John R. Turner[1], Jonathan W. King[2]

[1]School of Ocean Sciences, Bangor University, Menai Bridge, Anglesey, LL59 5AB, UK

[2] Centre for Applied Marine Sciences, Bangor University, Menai Bridge, Anglesey, LL59 5AB, UK

Corresponding author: nzwerschke01@qub.ac.uk

Supplementary Material 2:

SIMPER on macrofaunal assemblages between different densities of oyster abundance within different habitat types (bold).

	Percentage abundance		Diss/SD	Average cumulative % contribution
**Muddy Substratum**				
	Abundant	Common		
Barnacles	1.87	10.42	1.06	0.33
Ulva lactuca	6.76	3.78	0.64	0.52
Fucus spiralis	1.27	3.61	0.54	0.63
Mytilus edulis	1.46	1.92	0.87	0.72
Fucus vesiculosus	2.99	0.16	0.36	0.79
Littorina sp.	1.15	1.47	1.00	0.84
Ceramium rubrum	2.02	0.21	0.45	0.89
	Abundant	Absent		
Barnacles	1.87	5.10	0.96	0.20
Ulva lactuca	6.76	0.06	0.63	0.38
Fucus vesiculosus	2.99	1.08	0.43	0.48
Ascophyllum nodosum	0.02	4.51	0.39	0.58
Mytilus edulis	1.46	0.92	0.76	0.66
Littorina sp.	1.15	1.41	0.95	0.74
Fucus spiralis	1.27	0.78	0.51	0.80
Ceramium rubrum	2.02	0.01	0.45	0.86
Chondrus crispus	0.83	0.00	0.32	0.88

	Common	Absent		
Barnacles	10.42	5.10	1.18	0.41
Fucus spiralis	3.61	0.78	0.52	0.55
Ascophyllum nodosum	0.00	4.51	0.38	0.65
Mytilus edulis	1.92	0.92	0.82	0.75
Ulva lactuca	3.78	0.06	0.25	0.81
Littorina sp.	1.47	1.41	0.97	0.87

	Common	Super-abundant		
Barnacles	10.42	1.54	1.07	0.32
Fucus vesiculosus	0.16	4.87	0.64	0.45
Fucus spiralis	3.61	0.00	0.42	0.54
Ulva lactuca	3.78	0.49	0.34	0.62
Mytilus edulis	1.92	0.88	0.91	0.68
Chondrus crispus	0.02	1.77	0.63	0.75
Ceramium rubrum	0.21	1.82	0.69	0.80
Dictyota dichotoma	0.00	2.15	0.35	0.85
Littorina sp.	1.47	1.60	1.08	0.90

	Absent	Super-abundant		
Fucus vesiculosus	1.08	4.87	0.72	0.19
Barnacles	5.10	1.54	0.96	0.37
Ascophyllum nodosum	4.51	0.45	0.45	0.49
Chondrus crispus	0.00	1.77	0.63	0.57
Ceramium rubrum	0.01	1.82	0.69	0.64
Dictyota dichotoma	0.00	2.15	0.35	0.70
Littorina sp.	1.41	1.60	0.96	0.76
Mytilus edulis	0.92	0.88	0.84	0.81
Ulva lactuca	0.06	0.49	0.36	0.85
Fucus spiralis	0.78	0.00	0.40	0.88

Gravely Substratum

	Abundant	Absent		
Barnacles	4.10	3.60	1.12	0.18
Mytilus edulis	2.72	4.34	0.90	0.32
Fucus spiralis	3.04	0.83	0.60	0.42
Helminthocladia calvadosii	1.78	1.70	0.53	0.52
Fucus vesiculosus	3.46	1.46	0.40	0.62
Littorina sp.	1.70	0.68	1.03	0.69
Chondrus crispus	1.18	0.78	0.40	0.74

Pterocladiella cappillacea	0.09	0.76	0.46	0.78
Desmarestia viridis	0.27	0.56	0.40	0.80
Fucus serratus	0.19	0.76	0.34	0.83
Ostrea edulis	0.10	0.38	0.48	0.85
Red filamentous algae	0.43	0.01	0.31	0.87
Chlamys varia	0.28	0.41	0.31	0.89

	Common	Absent		
Helminthocladia calvadosii	4.28	1.70	0.66	0.14
Fucus vesiculosus	4.01	1.46	0.52	0.25
Barnacles	0.88	3.60	0.72	0.36
Mytilus edulis	0.51	4.34	0.66	0.45
Fucus serratus	2.47	0.76	0.54	0.53
Pterocladiella cappillacea	1.24	0.76	0.55	0.59
Chondrus crispus	1.85	0.78	0.70	0.65
Desmarestia viridis	1.35	0.56	0.57	0.71
Fucus spiralis	1.80	0.83	0.34	0.77
Ostrea edulis	0.51	0.38	0.63	0.80
Littorina sp.	0.45	0.68	0.74	0.83
Chlamys varia	0.65	0.41	0.43	0.85
Dictyota dichotoma	0.51	0.28	0.51	0.87
Green algae crust	0.42	0.12	0.56	0.89

Rocky Substratum

	Absent	Common		
Barnacles	7.28	7.05	0.91	0.26
Phymatolithon sp.	5.37	7.79	1.01	0.46
Osmundea pinnatifida	4.04	4.41	0.65	0.56
Fucus vesiculosus	3.37	4.59	0.59	0.67
Patella sp.	3.23	2.37	0.90	0.75
Catenella caespitosa	3.31	1.93	0.50	0.83
Littorina sp.	1.19	1.38	0.68	0.86
Mytilus edulis	1.28	0.71	0.58	0.90

	Absent	Abundant		
Barnacles	7.28	14.35	1.24	0.38
Phymatolithon sp.	5.37	2.44	0.80	0.52
Fucus vesiculosus	3.37	3.62	0.51	0.61
Patella sp.	3.23	1.71	0.87	0.69
Catenella caespitosa	3.31	2.31	0.39	0.76
Osmundea pinnatifida	4.04	1.05	0.51	0.83
Fucus serratus	1.46	0.00	0.18	0.85
Mytilus edulis	1.28	0.21	0.52	0.87

Valonia sp.	0.00	0.90	0.46	0.90

	Absent	Super-abundant		
Barnacles	7.28	12.90	1.31	0.36
Phymatolithon sp.	5.37	0.22	0.77	0.49
Patella sp.	3.23	2.27	0.96	0.57
Osmundea pinnatifida	4.04	0.14	0.43	0.63
Fucus vesiculosus	3.37	0.19	0.46	0.69
Catenella caespitosa	3.31	0.07	0.35	0.74
Mytilus edulis	1.28	1.21	0.75	0.79
Caulacanthus ustulatus	0.00	2.18	0.35	0.83
Fucus serratus	1.46	0.13	0.19	0.86
Littorina sp.	1.19	0.07	0.51	0.88

	Common	Abundant		
Barnacles	7.05	14.35	1.25	0.34
Phymatolithon sp.	7.79	2.44	0.87	0.53
Fucus vesiculosus	4.59	3.62	0.55	0.65
Osmundea pinnatifida	4.41	1.05	0.59	0.73
Catenella caespitosa	1.93	2.31	0.42	0.79
Patella sp.	2.37	1.71	0.82	0.85
Littorina sp.	1.38	0.36	0.58	0.88

	Common	Super-abundant		
Barnacles	7.05	12.90	1.31	0.32
Phymatolithon sp.	7.79	0.22	0.83	0.51
Fucus vesiculosus	4.59	0.19	0.48	0.59
Osmundea pinnatifida	4.41	0.14	0.52	0.66
Patella sp.	2.37	2.27	0.95	0.72
Mytilus edulis	0.71	1.21	0.69	0.77
Catenella caespitosa	1.93	0.07	0.44	0.81
Caulacanthus ustulatus	0.00	2.18	0.35	0.85
Littorina sp.	1.38	0.07	0.54	0.88

	Abundant	Superabundant		
Barnacles	14.35	12.90	1.34	0.42
Phymatolithon sp.	2.44	0.22	0.42	0.51
Fucus vesiculosus	3.62	0.19	0.38	0.60
Caulacanthus ustulatus	0.03	2.18	0.36	0.65
Valonia sp.	0.90	0.71	0.60	0.70
Mytilus edulis	0.21	1.21	0.66	0.75

Catenella caespitosa	2.31	0.07	0.23	0.79
Patella sp.	1.71	2.27	0.96	0.84
Osmundea pinnatifida	1.05	0.14	0.50	0.86
Ulva lactuca	0.03	0.82	0.39	0.88

Mussel bed Substratum

	Abundant	Absent		
Mytilus edulis	8.61	13.98	1.36	0.22
Chondrus crispus	4.88	9.86	1.29	0.40
Fucus vesiculosus	4.53	3.30	0.71	0.58
Ceramium rubrum	4.28	5.81	0.97	0.71
Ulva lactuca	2.19	3.63	1.11	0.78
Barnacles	3.11	3.53	1.34	0.83
Osmundea pinnatifida	1.18	0.89	0.52	0.86
Ulva intestinalis	0.64	1.28	0.90	0.89
	Abundant	Super-abundant		
Barnacles	3.11	77.83	7.47	0.54
Mytilus edulis	8.61	45.00	3.63	0.81
Chondrus crispus	4.88	9.71	2.54	0.87
Ceramium rubrum	4.28	4.14	1.14	0.91
	Abundant	Common		
Ulva lactuca	2.19	16.64	1.15	0.22
Chondrus crispus	4.88	17.44	2.09	0.44
Mytilus edulis	8.61	15.82	1.49	0.61
Ceramium rubrum	4.28	7.22	1.44	0.72
Fucus vesiculosus	4.53	0.00	0.48	0.79
Ulva intestinalis	0.64	3.41	1.39	0.83
Barnacles	3.11	4.49	1.29	0.88
	Absent	Super-abundant		
Barnacles	3.53	77.83	4.49	0.55
Mytilus edulis	13.98	45.00	1.86	0.79
Chondrus crispus	9.86	9.71	3.49	0.87
Ceramium rubrum	5.81	4.14	3.63	0.91
	Absent	Common		
Ulva lactuca	3.63	16.64	1.10	0.23
Chondrus crispus	9.86	17.44	1.28	0.44
Mytilus edulis	13.98	15.82	1.25	0.61

Ceramium rubrum	5.81	7.22	1.33	0.72
Fucus vesiculosus	3.30	0.00	0.57	0.79
Barnacles	3.53	4.49	1.19	0.83
Ulva intestinalis	1.28	3.41	1.19	0.88
	Super-abundant	Common		
Barnacles	77.83	4.49	9.61	0.53
Mytilus edulis	45.00	15.82	3.85	0.74
Ulva lactuca	0.37	16.64	1.04	0.85
Chondrus crispus	9.71	17.44	1.68	0.90
