# Supplementary material for: Limited impact of an invasive oyster on intertidal assemblage structure and biodiversity: the importance of environmental context and functional equivalency with native species
Source: Mar Biol. 2018 Apr 20;165(5):89. doi: 10.1007/s00227-018-3338-7 (PMC5910461; doi:10.1007/s00227-018-3338-7)
Supplement: Supplementary file 3 — Supplementary material 3 (PDF 105 kb) [file 227_2018_3338_MOESM3_ESM.pdf]

Marine Biology

Limited impact of an invasive oyster on intertidal assemblage structure and biodiversity: The importance of environmental context and functional equivalency with native species

Nadescha Zwerschke[1]*, Philip R. Hollyman[1], Romy Wild[1], Robin Strigner[1], John R. Turner[1], Jonathan W. King[2]

[1]School of Ocean Sciences, Bangor University, Menai Bridge, Anglesey, LL59 5AB, UK

[2] Centre for Applied Marine Sciences, Bangor University, Menai Bridge, Anglesey, LL59 5AB, UK

Corresponding author: nzwerschke01@qub.ac.uk

Supplementary Material 3:

Mean abundance (± SE) of species driving differences in macrofaunal assemblage structure at the different habitat types: gravel (G), muddy (M), mussel bed (MB) and rocky (R) at different oyster densities (absent, common, abundant and superabundant).

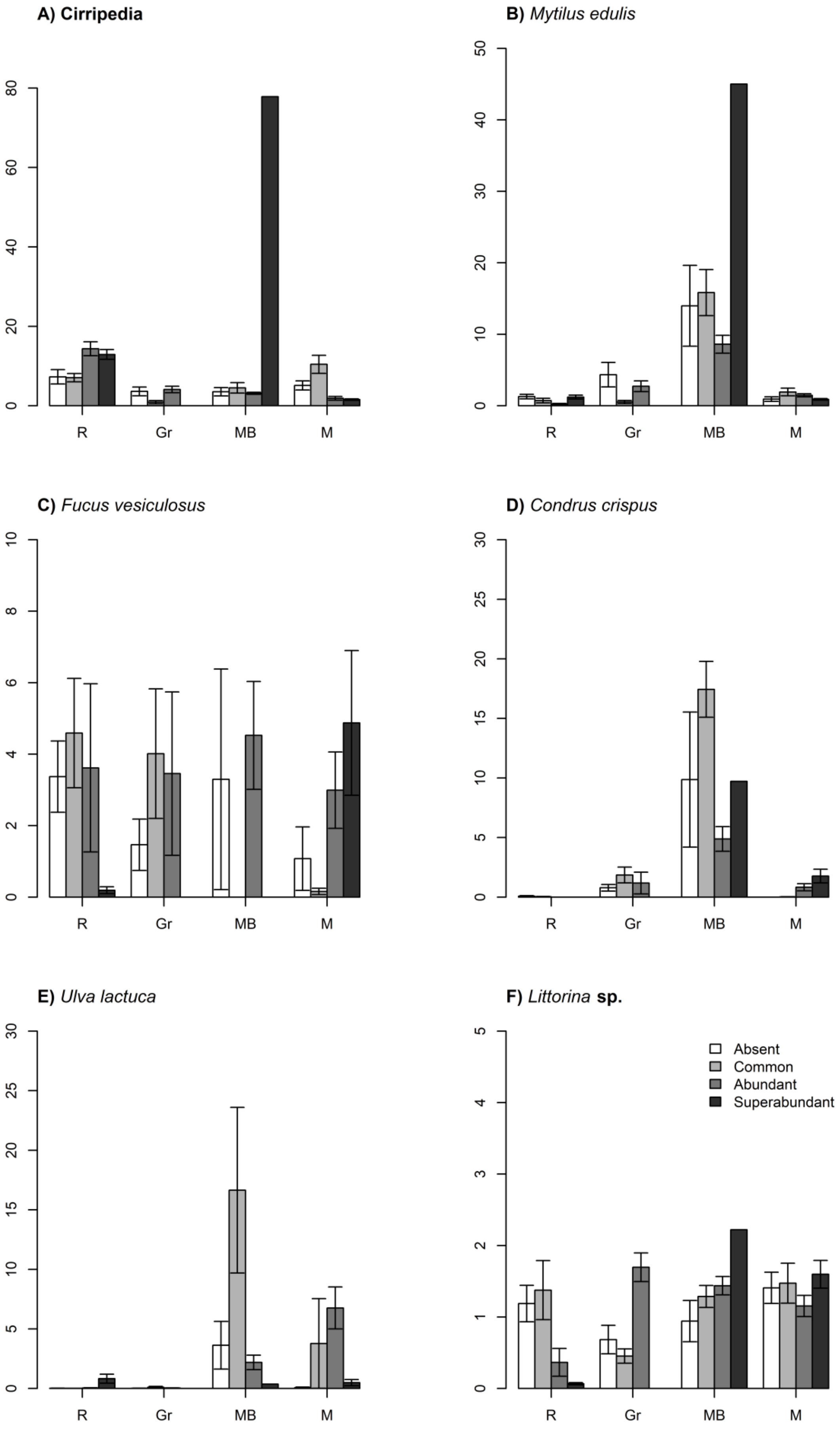
A) Cirripedia
B) *Mytilus edulis*
C) *Fucus vesiculosus*
D) *Condrus crispus*
E) *Ulva lactuca*
F) *Littorina* sp.
R
Gr
MB
M
0
20
40
60
80
10
30
50
2
4
6
8
5
15
25
1
3
Absent
Common
Abundant
Superabundant
