# Supplementary material for: Limited impact of an invasive oyster on intertidal assemblage structure and biodiversity: the importance of environmental context and functional equivalency with native species
Source: Mar Biol. 2018 Apr 20;165(5):89. doi: 10.1007/s00227-018-3338-7 (PMC5910461; doi:10.1007/s00227-018-3338-7)

Marine Biology

Limited impact of an invasive oyster on intertidal assemblage structure and biodiversity: The importance of environmental context and functional equivalency with native species

Nadescha Zwerschke[1]*, Philip R. Hollyman[1], Romy Wild[1], Robin Strigner[1], John R. Turner[1], Jonathan W. King[2]

[1]School of Ocean Sciences, Bangor University, Menai Bridge, Anglesey, LL59 5AB, UK

[2] Centre for Applied Marine Sciences, Bangor University, Menai Bridge, Anglesey, LL59 5AB, UK

Corresponding author: nzwerschke01@qub.ac.uk

Supplementary Material 4:

Uniquness of sites according to oyster abundances on the SACFOR scale for A) macrofauna and B) epifaunal assemblages.

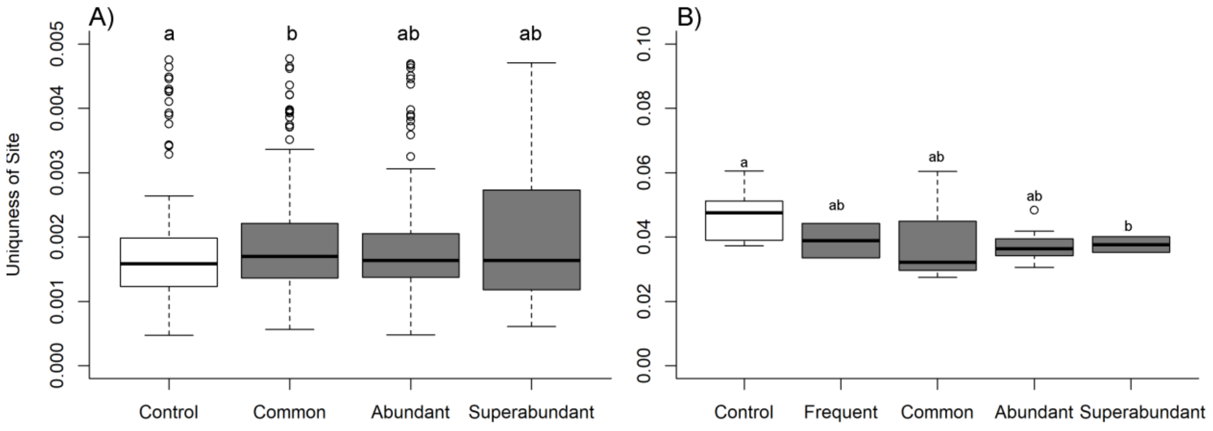

Supplement: Supplementary file 4 — Supplementary material 4 (PDF 193 kb) [file 227_2018_3338_MOESM4_ESM.pdf]
